# Regulatory mechanisms of Th9 cell differentiation

**DOI:** 10.3389/fimmu.2025.1650972

**Published:** 2025-09-05

**Authors:** Xingyue Liu, Ya Li, Wenwen Wu, Han Huang, Yanmei Hao, Chuanwang Song

**Affiliations:** ^1^ Anhui Province Key Laboratory of Immunology in Chronic Diseases, Bengbu Medical University, Bengbu, Anhui, China; ^2^ School of Laboratory Medicine, Bengbu Medical University, Bengbu, Anhui, China

**Keywords:** Th9 cells, IL-9, differentiation regulation, TGF-β, IL-4, epigenetic regulation, metabolic reprogramming

## Abstract

Th9 cells, a distinct subset of T helper cells, are defined by their production of IL-9. Th9 cells play a role in the development of various diseases by participating in mucosal immune responses, defending tissue barriers, and regulating inflammatory responses. For instance, Th9 cells contribute to inflammatory bowel disease by secreting IL-9, which damages the intestinal epithelial barrier. The effects mediated by Th9-derived IL-9 exhibit environment-dependent characteristics. In allergic asthma, IL-9 drives inflammation, while in specific tumor microenvironments, IL-9 can exert anti-tumor effects. Th9 cell differentiation is governed by a complex, multi-layered regulatory network. This network centers on the synergistic action of transforming growth factor-beta (TGF-β) and interleukin-4 (IL-4). Additionally, it involves multiple other mechanisms. These include exogenous signals such as IL-2 and IL-35; intrinsic transcription factors like the ATF-like protein BATF and PU.1; epigenetic modifications, including histone acetylation and DNA methylation; and metabolic reprogramming, such as glycolysis and lipid metabolism, among others. This review systematically summarizes the regulatory mechanisms governing Th9 cell differentiation. It elucidates these mechanisms and reveals potential therapeutic targets, including transcription factors such as PU.1, IRF4, and BATF. This work paves the way for the development of Th9-related immunotherapies.

## 1 Introduction

As fundamental components of adaptive immunity, T cells play a key role in maintaining immune homeostasis and mediating cellular immunity. CD4^+^ T cell activation is initiated when their T cell receptors (TCRs) recognize antigen peptide-MHC class II complexes presented by antigen-presenting cells (APCs). This recognition, combined with the integration of co-stimulatory signals, triggers the activation cascade. After activation, CD4^+^T cells differentiate into distinct helper T (Th) cell subsets by expressing lineage-specific transcription factors, driven by a specific cytokine microenvironment. Among the various T cell subsets, Th9 cells have attracted considerable attention due to their unique biological properties (1, 2). Studies have shown that patients with allergic asthma have significantly higher numbers of Th9 cells than healthy individuals (3). Th9 cells secrete IL-9 to enhance the production of type 2 cytokines IL-4, IL-5, and IL-13, induce airway mucus secretion, and promote eosinophil recruitment, amplifying the asthmatic inflammatory response. The number of Th9 cells in patients with rheumatoid arthritis (RA) is also significantly higher than that in normal individuals (4). These findings underscore the pivotal role of Th9 cells in disease pathogenesis. Therefore, it is essential to understand the intricate regulatory network governing Th9 cell differentiation to elucidate their contribution to disease and develop targeted therapeutic strategies.

**Figure 1:**
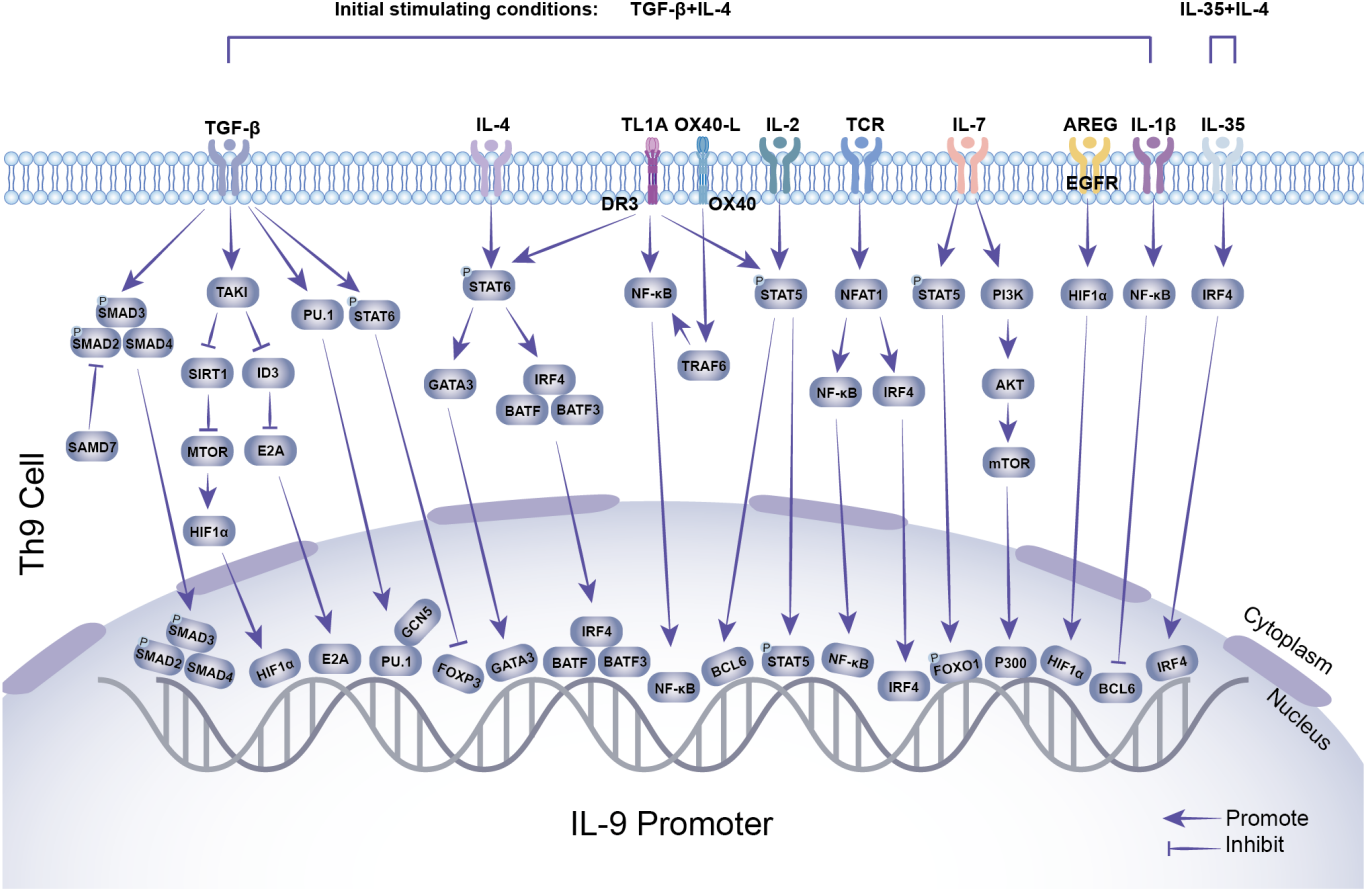
Transcriptional regulation of Th9 cell differentiation. TGF-β, IL-4, TL1A, OX40, IL-2, TCR, and other stimuli induce the expression of downstream transcription factors, including SMAD2/SMAD3/SMAD4, HIF-1α, E2A, PU.1, FOXP3, GATA3, BATF, BATF3, IRF4, NF-κB, BCL6, STAT5, and FOXO1. These transcription factors interact with the IL-9 promoter, while GCN5 and P300 enhance histone modifications, synergistically promoting IL-9 expression and secretion.

As shown in **Figure 1** and **Tables 1**–**3**, Th9 cell differentiation is regulated by multiple factors. The process initiates with fundamental T cell receptor (TCR) recognition of antigen and co-stimulatory signals. These signals are mediated by molecules and pathways, such as NFAT1/CBP/P300 and NF-κB, which establish the foundation for subsequent lineage commitment. The synergistic action of the exogenous cytokines TGF-β and IL-4 then serves as the key signal that initiates the differentiation of naïve CD4^+^ T cells into the Th9 lineage (7, 38). TGF-β and IL-4 promote Th9 differentiation by suppressing FOXP3 expression and activating the STAT6/GATA3 axis in activated naïve CD4^+^ T cells. Endogenous transcription factor BATF is a key transcription factor in the early development of Th9 cells, which promotes Th9 cell differentiation by forming a complex with IRF4 (16). As a downstream transcription factor of TGF-β signaling, PU.1 is a core regulatory factor for both the differentiation and functional exertion of Th9 cells. PU.1 binds to the IL-9 promoter, regulates chromatin accessibility to enhance IL-9 transcription, thereby promoting the directional differentiation of Th9 cells (20). Epigenetic regulation, including histone acetylation and DNA methylation, finely regulates the expression of the key cytokine IL-9 and the core transcription factor PU.1 in Th9 cells by altering chromatin accessibility and gene expression levels. Hypoxia-inducible factor-1α (HIF-1α) is a key molecule linking metabolism and transcriptional regulation. HIF-1α supplies the necessary energy for Th9 cell differentiation by promoting metabolic pathways such as glycolysis (30). Moreover, it functions as a signaling molecule to regulate transcriptional programs. This reflects the close association between metabolism and Th9 cell differentiation. This review systematically summarizes the latest research progress in the field of Th9 cell subset differentiation, laying a theoretical foundation for elucidating the mechanisms of Th9 cell differentiation and developing novel immunotherapies for related diseases.

**Table 1:** The exogenous regulation network of Th9 differentiation.

Factor	Mechanism	Effect on Th9 Differentiation	Reference
TGF-β	Acts via SMAD-dependent and -independent pathways to regulate gene expression	Promotes	(5)
IL-4	Synergizes with TGF-β to activate STAT6 phosphorylation and induce GATA3 expression.	Promotes	(6)
TGF-β + IL-4	Synergistically inhibits FOXP3 and activates PU.1	Promotes	(7)
TCR	Activates NFAT1/CBP/p300, enhancing chromatin accessibility at the IL-9 promoter	Promotes	(8)
TL1A-DR3	Activates the IL-2/STAT5 signaling pathway and increases production of TGF-β, IL-4, and PU.1	Promotes	(8, 9)
OX40	Recruits TRAF6 to activate the NF-κB pathway	Promotes	(10)
IL-2	Activates STAT5 and inhibits BCL6	Promotes	(11, 12)
IL-1β	Works with IL-4 to activate NF-κB and inhibit BCL6, substituting for TGF-β	Promotes	(13)
TLR2	Enhances Th9 differentiation by upregulating BATF and PU.1.	Promotes	(14)
IL-35	Activates IRF4 and substitutes for TGF-β	Promotes	(15)

TGF-β, transforming growth factor β; IL-4, interleukin-4; TCR, T-cell receptor; TL1A-DR3, TNF-like ligand 1A and death receptor 3; OX40, TNF receptor superfamily member 4; IL-2, interleukin-2; IL-1β, interleukin-1 β; TLR2, Toll-like receptor 2; IL-35, interleukin-35.

**Table 2:** The endogenous regulation network of Th9 differentiation.

Factor	Mechanism	Effect on Th9 Differentiation	Reference
BATF	Forms complex with IRF4 on IL-9 promoter	Promotes	(16, 17).
IRF4	Binds to the IL-9 promoter to enhance IL-9 transcription	Promotes	(18, 19)
PU.1	Inhibits GATA3 and recruits GCN5/p300 histone acetyltransferases to enhance IL-9 promoter acetylation	Promotes	(20, 21)
ETV5/ERG	Recruits p300 to catalyze histone acetylation and enhances IL-9 transcription.	Promotes	(22, 23)
FOXO1	Promotes the expression of IRF4	Promotes	(24, 25)
FOXP2	Disrupts BATF/IRF4 complexes via downregulation	Inhibits	(26)
FOXP3	Foxp3 binds to GATA3 to inhibit GATA3 from activating the IL-9 promoter	Inhibits	(7)
LINC00240	Inhibits DNMT1 via miR-155-5p sponge	Promotes	(27)
miR-143/145	Inhibits NFATc1 expression	Inhibits	(28)
miR-493-5p	Inhibits FOXO1 expression	Inhibits	(29)

BATF, basic leucine zipper transcription factor ATF-like; IRF4, interferon regulatory factor 4; PU.1, Spi-1 proto-oncogene; ETV5/ERG, ETS variant 5 and ETS-related gene; FOXO1, forkhead box protein O1; FOXP2, forkhead box P2; FOXP3, forkhead box P3; LINC00240, long intergenic non-protein coding RNA 240; miR-143/145, microRNA-143/microRNA-145; NFATc1, nuclear factor of activated T-cells cytoplasmic 1; miR-493-5p, microRNA-493-5p.

**Table 3:** The metabolic regulation network of Th9 differentiation.

Factor	Mechanism	Effect on Th9 Differentiation	Reference
HIF-1α	Enhances IL-9 transcription through glycolysis and the EGFR signaling pathway	Promotes	(30, 31)
Succinate	Stabilizes the HIF-1α	Promotes	(31)
α-KG	Promotes the degradation of HIF-1α	Inhibits	(31)
Polyamines	Enhances the expression of GATA3	Promotes	(32)
Butyrate	Induces the expression of FOXP3	Inhibits	(33)
vitamin D	Downregulates PU.1; inhibits histone acetylation and TLR2-IL-33 pathway	Inhibits	(34, 35)
Piezo1	Regulates the HIF1α-IL-9 axis via mitochondrial oxidative phosphorylation	Promotes	(36)
Sphingosylphosphorylcholine	Elevates mitochondrial ROS to activate SMAD3/STAT5/β-catenin, promoting Th9 differentiation	Promotes	(37)

HIF-1α, α hypoxia-inducible factor 1-alpha; α-KG, Alpha-ketoglutarate; ACC1 inhibitor, acetyl-CoA carboxylase 1 inhibitor; Piezo1, Piezo-type mechanosensitive ion channel component 1.

## 2 Discovery and establishment of Th9 cells

After activation, naïve CD4^+^ T cells differentiate into distinct Th cell subsets. This process is driven by TCR signals and cytokines, which activate lineage-defining transcription factors. These transcription factors then determine the cytokine secretion and immune functions of each subset. Th2 cell differentiation is primarily driven by the IL-4/STAT6 signaling pathway and regulated by the transcription factor GATA3 (39–41). IL-4, IL-5, and IL-13 secreted by Th2 cells mediate IgE production and eosinophil recruitment, and are crucial in allergic reactions and anti-helminth immunity (42, 43).

Th17 cell differentiation depends on the TGF-β/IL-6 signaling pathway and is regulated by the transcription factor RORγt (44, 45). Th17 cells secrete IL-17 and IL-22, which play a role in defending against extracellular bacterial and fungal infections (46, 47). Regulatory T cells (Tregs) differentiate in response to TGF-β and IL-2 and are regulated by the transcription factor Foxp3 (48, 49). Treg cells maintain immune tolerance and regulate immune response intensity by secreting TGF-β and IL-10 (50–52).

Th9 cells are a subpopulation of CD4^+^ helper T cells that secrete IL-9. Although early studies initially categorized Th9 cells as a subset of Th2 cells due to their shared IL-9 production capacity, a 2008 study by Veldhoen et al. found that TGF-β induced the transformation of Th2 cells into IL-9-producing helper T cells, which did not express transcription factors characteristic of Th1 (T-bet), Th17 (RORγt), or regulatory T cells (FOXP3) (6). In the same year, Dardalhon et al. showed that TGF-β and IL-4 work together to decrease Foxp3 expression and differentiate naïve CD4^+^ T cells into a distinct subset of T cells that secreted IL-10 and IL-9 (7). Phenotypically, Th9 cells express CD183 (CXCR3), CD193 (CCR3), and CD196 (CCR6), but lack the Th2-characteristic markers CD194 (CCR4) or CD294 (CRTH2) (53, 54). As well as demonstrating that Th9 cells are a distinct subgroup (55–57), these studies also highlighted the crucial regulatory role that synergistic TGF-β and IL-4 signaling play in Th9 cell differentiation. Subsequent studies have further shown that this synergistic action collectively shapes the functional diversity of Th9 cells through multilayered mechanisms, including exogenous signaling, endogenous transcription factor networks, epigenetic modifications, and metabolic regulation.

## 3 Regulation of antigen recognition signaling

Th9 cell differentiation and IL-9 secretion are significantly regulated by activation of TCR and its co-stimulatory signals. Following TCR activation, nuclear factor of activated T cells 1(NFAT1) attracts the histone acetyltransferases CBP/P300, thereby enhancing chromatin accessibility and facilitating the recruitment of NF-κB to the IL-9 promoter to induce transcription (8). In addition, TCR promotes Th9 differentiation by upregulating the expression of interferon regulatory factor 4 (IRF4) (18, 58). Similarly, the TNF family protein TL1A binds to Death Receptor 3 (DR3) to activate the IL-2/STAT5 signaling pathway and increase production of TGF-β, IL-4, and PU.1, which collectively induce Th9 differentiation (8, 9). Recent studies have revealed that activation of TNF receptor superfamily member 4 (OX40) promotes the differentiation of CD4^+^ T cells toward Th9 cells by inhibiting the differentiation of both induced regulatory T cells (iTregs) and Th17 cells (10, 59). Mechanistically, OX40 has been shown to induce Th9 cells through activation of the NF-κB signaling pathway via TNF receptor-associated factor 6 (TRAF6) (10).

## 4 Exogenous signal regulation

Th9 cell differentiation is precisely regulated by a range of exogenous signals, with TGF-β and IL-4 acting as the primary initiation signals. TGF-β and IL-4 work together with T cell receptors (TCRs), IL-2, and Toll-like receptors (TLRs) to drive Th9 differentiation. In addition, several regulatory pathways have been shown to operate independently of TGF-β and IL-4, highlighting the complexity and diversity of the Th9 differentiation network.

### 4.1 TGF-β and IL-4 signaling

As a crucial component of the transforming growth factor-β superfamily, the TGF-β controls Th9 cell development via dual mechanisms that are SMAD-dependent and SMAD-independent (6, 60, 61). In the SMAD-dependent pathway, TGF-β binds to its receptors to promote a SMAD2/3/4 heterotrimeric complex, which translocates to the nucleus, where it binds to transcription factors, thereby controlling the expression of target genes and subsequently promoting Th9 development (62). SMAD7 inhibits this process by preventing the SMAD complex from binding to DNA (63). In the non-SMAD pathway, TGF-β and IL-4 synergistically activate TAK1, downregulate ID3 expression, promote the binding of E2A/GATA binding protein 3 (GATA3) to the IL-9 promoter, and increase the transcriptional activity of IL-9 (5). Furthermore, suppression of sirtuin 1 (SIRT1) deacetylase expression by TAK1 leads to increased IL-9 secretion and glycolytic activity, whereas overexpression of exogenous SIRT1 has the opposite effect (64).

Signal transducer and activator of transcription 6 (STAT6) and GATA3 are key regulators of Th9 cell differentiation (6, 7, 65). IL-4 induces GATA3 expression by activating STAT6 phosphorylation and driving Th2 cell differentiation (6). However, when IL-4 cooperates with TGF-β, it suppresses TGF-β-induced generation of Foxp3^+^, Tregs through the IL-4-STAT6-GATA3 axis while simultaneously downregulating the production of Th2-type cytokines (e.g., IL-4, IL-5, IL-13). This process consequently induces the generation of IL-9-secreting Foxp3^-^ effector T cells, ultimately promoting Th9 cell differentiation (6). Paradoxically, IL-4 negatively regulates Th9 differentiation under specific conditions.IL-4-induced cytokine-inducible SH2-containing protein (CIS) suppresses activation of STAT3, STAT5, and STAT6, thereby inhibiting Th9 cell differentiation. Consequently, deficiency of CIS in T cells significantly enhances both Th2 and Th9 cell differentiation. This indicates that IL-4 inhibits Th9 differentiation by inducing CIS (66).

### 4.2 Cytokines

Cytokines play pivotal roles in Th9 cell differentiation through complex and diverse regulatory mechanisms, exhibiting both promoting and inhibitory effects. Among cytokines that enhance Th9 differentiation, IL-2 serves as a critical regulator. IL-2 controls Th9 differentiation and IL-9 secretion through two distinct mechanisms, which involve either the activation of STAT5 or the inhibition of B-cell lymphoma 6 (BCL6). IL-2-mediated phosphorylation of STAT5 results in the direct binding of STAT5 to the IL-9 promoter and subsequent IL-9 transcription, whereas a shortage or blockage of IL-2 inhibits IL-9 synthesis (11, 12). IL-2 can also promote Th9 differentiation by suppressing expression of the transcriptional repressor BCL6, which competes with STAT5 to bind to the IL-9 promoter (11). Jiang et al. elucidated that TNF-α comprehensively enhances Th9 cell differentiation, survival, and proliferation through the TNFR2-STAT5 and NF-κB signaling pathways (67). Furthermore, cytokines including IL-36γ from the IL-1 family (68), IL-1 (69), IL-2 (11, 70), IL-6 (71), IL-10 (7), IL-23 (72), IL-25 (73), IL-33 (74), IFNα/β (75), and TSLP (76, 77) contribute to Th9 cell induction. However, their precise molecular mechanisms require further elucidation. Conversely, certain cytokines suppress Th9 cell differentiation. Murugaiyan et al. have demonstrated that IFN-γ directly inhibits Th9 cell differentiation through STAT1-mediated signaling; moreover, it has induced dendritic cells to produce IL-27, which has partially suppressed Th9 cell development through STAT1 and T-bet (78).

### 4.3 TLRs

As receptors that recognize innate immunological patterns, Toll-like receptors (TLRs) control T cell growth and have a direct impact on Th9 differentiation (79). Studies have shown that in the presence of TGF-β and IL-4, the activation of TLR2 promotes Th9 cell differentiation by upregulating the transcription factors BATF and PU.1 (14). Moreover, a negative correlation between the activity of TLR2 and serum vitamin D levels has been observed, suggesting that vitamin D may negatively regulate Th9 differentiation by inhibiting the TLR2 pathway (34).

### 4.4 Regulatory mechanisms independent of TGF-β and IL-4

Although some interleukins, such as IL-35 and IL-1β, can functionally replace TGF-β under certain circumstances, they still need to work in concert with IL-4 to induce Th9 differentiation. For example, IL-35 has been shown to promote Th9 differentiation through activation of IRF4 via the IL-12Rβ2/GP130 receptor. However, the lack of synergistic effects between TGF-β1 and IL-35 at optimal doses indicates that they probably share partial downstream signaling pathways. In the presence of IL-4, IL-35 can replace TGF-β1 to induce the differentiation of naïve CD4^+^ T cells into Th9 cells, although the underlying mechanisms remain unclear and require further elucidation (15). Other studies have shown that when combined with IL-4, IL-1β can act through the IL-1R signaling pathway to replace TGF-β and promote Th9 differentiation (13). Initiating synergistic effects requires the presence of TGF-β or its alternative components, whereas IL-4 plays a crucial role in Th9 differentiation. Surprisingly, TGF-β and IL-4 are not required for IL-33 to promote Th9 differentiation. For example, dectin-1 signaling in dendritic cells has been shown to promote the production of IL-33, which then acts through its suppression of tumorigenicity 2 (ST2) receptor to induce differentiation of CD4^+^ T cells into Th9 cells, which produce IL-9 (80).

## 5 Endogenous regulation

The fate of Th9 cells depends not only on exogenous signals but also on endogenous regulatory networks. The endogenous regulatory network of Th9 cell development is provided by the synergistic signals of TGF-β and IL-4, which regulate key transcription factors like PU.1, IRF4, and BATF, leading to epigenetic changes like histone acetylation. Several levels of molecular regulation mediate this process to guarantee accurate Th9 cell differentiation.

### 5.1 Transcription factor BATF

BAFT, a key transcription factor for Th9 development, binds to the IL-9 promoter and controls its production by forming a complex with IRF4 downstream of the TGF-β/IL-4 signaling pathway. While co-expression of BAFT with IRF4 synergistically increases IL-9 production, BATF deficiency dramatically decreases IL-9 production (16). TL1A stimulation has been shown to upregulate Basic leucine zipper ATF-like transcription factor 3 (BATF3), a homolog of BATF, which combines with BATF and IRF4 to form a complex that enhances IL-9 promoter binding and dramatically increases IL-9 secretion (17).

### 5.2 Transcription factor IRF4

IRF4, a transcription factor that is essential for Th9 differentiation, drives transcription by binding directly to the IL-9 promoter. IRF4-depleted CD4^+^ T cells are unable to differentiate into Th9 cells, highlighting the importance of IRF4 in this process (18). Meanwhile, TGF-β and IL-4 have been shown to promote the conversion of Th2 to Th9 cells through the SMAD3/SMAD4 signaling pathway and induction of IRF4 expression (19). Interferon Regulatory Factor 8 (IRF8) and IRF4 exhibit structural similarities, and have been shown to work together with BATF and PU.1 to form a transcriptional complex that binds to the IL-9 promoter to increase IL-9 expression (81).

### 5.3 ETS transcription factor family

PU.1 is a fundamental member of the ETS transcription factor family and the first transcription factor to be found to play an essential role in the specific production of IL-9 in Th9 cells. Studies indicate that PU.1-deficient mice exhibit reduced IL-9 expression. Conversely, PU.1 confers upon Th9 cells the capacity for robust IL-9 production (20). PU.1 suppresses Th2-specific gene expression downstream of TGF-β and IL-4 signaling through inhibition of GATA3 (20). Furthermore, PU.1 has been shown to form a complex with the histone acetyltransferase General Control Non-derepressible 5 (GCN5), which increases histone H3/H4 acetylation levels in the IL-9 promoter region, thereby markedly increasing transcriptional activity and chromatin accessibility (21, 82). Another member of the ETS family, ETS variant transcription factor 5 (ETV5), promotes histone H3K27 and H4K16 acetylation by recruiting the histone acetyltransferase P300, thereby enhancing IL-9 transcription (22). ERG has also been shown to combine with PU.1 and ETV5 to produce a complex that increases the transcriptional activity and binding effectiveness of the IL-9 promoter (23).

### 5.4 FOX family of transcription factors

Members of the FOX family are involved in regulating the differentiation of Th9 cells. One member, Forkhead box O1 (FOXO1), is a key positive regulatory factor that binds directly to the IL-9 promoter to boost its transcription. FOXO1 has also been shown to promote IRF4 expression, which greatly increases the efficiency of Th9 differentiation (24, 25). In contrast, Forkhead box P1 (FOXP1) has been identified as a negative regulator of Th9 differentiation since it inhibits IL-9 production and prevents Th9 differentiation. In a recent study, IL-7 signaling was found to induce FOXO1 to displace FOXP1 in the nucleus and promote IL-9 production (83). Forkhead box O4 (FOXO4), another member of the FOX family, has also been shown to promote Th9 differentiation (83). Conversely, Th9 differentiation is suppressed by Forkhead box P2 (FOXP2) and Forkhead box P3 (FOXP3). FOXP2 has been shown to adversely affect Th9 differentiation by downregulating the expression of BATF and IRF4 and subsequently destabilizing the core transcriptional complex necessary for Th9 development (26). Th9 differentiation is further negatively regulated by the binding of FOXP3 to GATA3, which inhibits the transcription of Th9-associated genes (7).

### 5.5 Epigenetic regulation

Covalent histone modifications and DNA methylation are two of the many ways that epigenetic regulation affects Th9 cell development. At the level of histone modifications, Chromobox protein homolog 4 (Cbx4) increases the transcriptional activity of the Hypoxia-inducible factor-1 alpha (HIF-1α) protein, stabilizing it through SUMOylation, and thus promoting IL-9 expression (84). IL-7 induces the histone acetyltransferase p300 through the STAT5-PI3K-AKT-mTOR pathway, thereby promoting histone acetylation at the IL-9 promoter region and activating transcription (83). By sponging miR-155-5p, the long non-coding RNA LINC00240 has been shown to control DNMT1-mediated PU.1 promoter methylation at the DNA methylation level. While high expression of LINC00240 inhibits this process, low expression alleviates PU.1 inhibition and promotes Th9 differentiation by lowering methylation levels (27).

### 5.6 Non-coding RNA

The lncRNA HOTAIRM1 has been shown to promote Th9 differentiation by competitively binding to miR-148a-3p and reducing its transcriptional repression on IRF4 (85). In contrast, miR-143/145 expression levels are significantly inversely correlated with Th9 differentiation, with miR-143/145 reducing the IL-9 transcriptional pathway by targeting and suppressing Nuclear factor of activated T-cells cytoplasmic 1 (NFATc1) expression (28). Finally, the tumor suppressor gene miR493-5p inhibits Th9 cell development by inhibiting the expression of FOXO1 (29).

## 6 Metabolic regulatory network

Recent studies have elucidated the pivotal roles of metabolites in Th9 cell differentiation. Metabolic pathways not only supply energy and biosynthetic intermediates for cells but also influence the differentiation fate of Th9 cells by modulating key signaling pathways and transcription factors.

### 6.1 Metabolic reprogramming

Distinct metabolic characteristics are closely associated with Th9 differentiation. Extracellular ATP provides energy support for Th9 development by activating the purinergic receptor mTOR-HIF-1α signaling axis, which in turn induces the generation of NO (30). Furthermore, interactions between amphiregulin and the EGFR strengthen the differentiation process by increasing the ability of HIF-1α to bind to the IL-9 promoter (31). Notably, when compared to other Th subsets, Th9 cells show significantly more glycolytic dependence. In addition to reducing glycolytic capacity, HIF-1α deficiency also interferes with the tricarboxylic acid cycle, pentose phosphate pathway, and fatty acid metabolic network (30). Dysregulation of these metabolic pathways severely disrupts the energy homeostasis of Th9 cells, leading to significant inhibition of their differentiation.

### 6.2 Metabolites

The metabolic microenvironment further regulates Th9 differentiation through metabolitic homeostasis. While succinate stabilizes HIF-1α through succinylation modifications to promote Th9 differentiation, α-ketoglutarate (α-KG) suppresses Th9 differentiation by promoting degradation of HIF-1α (31). Butyrate from gut bacteria and polyamines from the host have opposing effects. Butyrate reduces IL-9 synthesis by activating FOXP3 (33), whereas polyamines positively regulate IL-9 production by increasing GATA3 expression (32). The regulatory network of metabolites provides novel treatment targets for metabolically-based immunomodulatory methods by highlighting the critical role of microenvironmental metabolic homeostasis on Th9 differentiation.

### 6.3 Lipid metabolism and the physical microenvironment

Lipid metabolism plays a role in regulating Th9 differentiation through two independent pathways. First, Th9 differentiation and IL-9 production are markedly increased by the expression of Acetyl-CoA carboxylase 1 (ACC1), a fatty acid synthase, and 3 - Hydroxy - 3 - methylglutaryl - CoA reductase (HMGCR), a rate-limiting enzyme in cholesterol synthesis (86). Second, Th9 differentiation is negatively regulated by inhibiting ACC1-mediated fatty acid synthesis via the retinoic acid receptor-TGF-β-SMAD signaling axis. The crucial role of lipid metabolic homeostasis in Th9 differentiation is highlighted by the fact that exogenous oleic acid reverses the effects of ACC1 inhibitors or lipid uptake blockers, which markedly increase IL-9 release (87, 88). Furthermore, the mechanosensory receptor Piezo1 regulates HIF-1α signaling by modulating mitochondrial oxidative phosphorylation, while its functional deficiency suppresses Th9 differentiation (36). These studies reveal a novel mechanism through which the physical microenvironment governs Th9 differentiation.

### 6.4 Metabolic intervention molecules

Vitamin D, a crucial nutrient, inhibits Th9 differentiation by downregulating the expression of transcription factors including PU.1, IRF4, and BATF, and decreasing histone acetylation levels at the IL-9 promoter region (35). In contrast, sphingosylphosphorylcholine activates SMAD3, STAT5, and β-catenin signaling pathways by increasing mitochondrial reactive oxygen species and positively regulating Th9 differentiation (37). These studies show that the metabolic network is closely associated with transcription factor activity and epigenetic modifications. However, since recent studies have focused on single metabolic pathways, future studies need to analyze the mutual effects between different metabolic axes to provide a theoretical basis for the development of precision therapies targeting metabolic pathways.

## 7 The application of omics technologies

The development of omics technologies has enabled further exploration of the differentiation mechanisms and functional heterogeneity of Th9 cells. Single-cell transcriptome analysis revealed functionally distinct Th9 subpopulations based on CD96 expression; the CD96low Th9 subpopulation exhibits higher IL-9 expression and greater inflammatory potential, indicating that CD96 negatively regulates Th9 cytokine production and inflammatory effects (89). An integrated analysis of epigenomics (ATAC-seq and ChIP-seq) and transcriptomics (RNA-seq) revealed that the chromatin state around the IL-9 gene locus undergoes dynamic changes during Th9 differentiation. Specifically, it opens during differentiation and closes upon return to the resting state. This process involves histone modification remodeling, and depends on the transcriptional regulation of STAT5 and STAT6. These changes contribute to the bystander activation and maintenance of lineage stability in Th9 cells (90). Retinoic acid (RA) suppresses Th9 differentiation and associated inflammation. It acts through its receptor RARα to downregulate IL-9 and related gene expression, interfere with chromatin accessibility, and disrupt transcription factor binding (91). Epigenomic DNA methylation analysis showed inconsistent changes in the methylation patterns of Th9 differentiation markers. This suggests DNA methylation may not be a primary regulator of Th9 differentiation (92). These omics studies reveal the complex regulatory network governing Th9 cell differentiation. They provide an important foundation for a deeper understanding of Th9 biology and the mechanisms of related diseases.

## 8 Therapeutic prospects of Th9 cells in diseases

Th9 cells influence the progression of various diseases. PU.1-mediated IL-9 secretion by Th9 cells disrupts the intestinal barrier, contributing to disease exacerbation in ulcerative colitis (82). Adoptive transfer of Th9 cells significantly inhibits melanoma progression. This anti-tumor effect relies on IL-9-mediated tumor cytotoxicity and activation of CD8^+^ T cells (93, 94).In children with methicillin-resistant Staphylococcus aureus (MRSA) pneumonia, miR-155 promotes Th9 cell differentiation by silencing the deacetylase Sirtuin 1 (SIRT1), exacerbating pulmonary inflammation (95). Thus, Th9 cells have emerged as a potential therapeutic target for various diseases (**Table 4**). Studies have shown that Dioscin from Dioscorea nipponica Th9 cell differentiation by suppressing TGF-β and IRF4 in the tumor necrosis factor-like ligand 1A-death receptor 3 (TL1A-DR3) pathway, providing a new insight for the treatment of RA (99). These findings establish the therapeutic value of targeting Th9 cells and their associated signaling pathways, providing novel approaches for precision treatment of Th9-related diseases.

**Table 4:** Therapeutic modulators of Th9 cell differentiation.

Factor	Mechanism	Effect on Th9 Differentiation	Effect on diseases	Reference
TXA2	Activates the TP receptor, induces p38 MAPK activation, promotes binding of transcription factors NFE2 and PBX1 to the IL-9 promoter, and inhibits IL-9 transcription	Inhibits	Attenuates allergic lung inflammation	(96)
NO	Increases p53-mediated IL-2 production, STAT5 phosphorylation and IRF4 expression	Promotes	Aggravates airway inflammation	(97)
DTA-1	Enhances the differentiation of CD4^+^T cells into Th9 cells through the TRAF6-NF-κB pathway	Promotes	Promotes tumor-specific cytotoxic T lymphocyte responses	(98)
Retinoic Acid	Targets the extended IL9 locus and broadly modified the Th9 epigenome through RARα	Inhibits	Suppresses lung inflammation	(91)
DDN	Targets TGF-β and IRF-4 in the TL1A/DR3 pathway	Inhibits	Alleviating inflammation in arthritis models	(99)

TXA2, thromboxane A2; NO, nitric oxide; DTA-1, GITR agonistic antibody, GITR, glucocorticoid—induced tumor necrosis factor receptor; DDN, Dioscin from Dioscorea nipponica.

## 9 Discussion

This review synthesizes the multilayered regulatory architecture governing Th9 cell differentiation, encompassing exogenous signals, transcriptional networks, epigenetic dynamics, and metabolic reprogramming. These interconnected layers collectively determine Th9 differentiation fate through context-dependent mechanisms.

Current discoveries of molecules and pathways regulating Th9 differentiation offer foundational insights for clinical translation. For instance, vitamin D suppresses Th9 differentiation by downregulating the transcription factor PU.1. This mechanism identifies a potential therapeutic target for inflammatory diseases characterized by Th9 overactivation (35). Combining Th9 cells with anti-PD-1 therapy enhances antitumor activity through synergistic effects (100). The discovery of such therapeutic targets demonstrates the translational potential of Th9 differentiation regulatory mechanisms. Future studies should integrate single-cell sequencing and gene editing technologies to decipher the underlying mechanisms controlling Th9 differentiation, enabling effective intervention strategies for Th9-related diseases.

Current research exhibits limitations regarding the dual regulatory functions of specific molecules in Th9 differentiation. Low-concentration IL-4 promotes differentiation via STAT6 signaling, serving as an early-stage driver (65). Conversely, high-concentration IL-4 indirectly suppresses Th9 differentiation through CIS induction (66). This bidirectional regulation may be modulated by multiple variables, including microenvironmental context, cytokine concentration, and temporal dynamics. Whereas existing studies remain largely limited to single-concentration paradigms and static observations. A systematic dissection of the dose-time-pathway axis is therefore imperative. Secondly, the specific transcription factors involved in Th9 cell differentiation have not yet been identified, which poses challenges for the precise regulation of Th9 cell differentiation. Furthermore, the fate plasticity of Th9 differentiation remains unclear. When the inflammatory microenvironment changes, will Th9 cells transdifferentiate into other helper T cell subsets? Most current studies have been conducted in animal models, and the role of Th9 cells in diseases still requires further verification in human samples. Moreover, the interaction mechanisms between Th9 and other helper T cell subsets remain elusive. It is poorly defined whether Th9 cells collaborate with or compete against other helper T cells during immune responses, and how such dynamic interplays influence disease progression. Answering these questions will improve our understanding of the biological characteristics of Th9 cells and help us develop more effective therapeutic strategies.
